# Using modern concepts to engage the world in an annual public health campaign

**DOI:** 10.1186/2047-2994-4-S1-P106

**Published:** 2015-06-16

**Authors:** C Kilpatrick, A Agostinho, J Storr, E Kelly, B Allegranzi, D Pittet

**Affiliations:** 1 World Health Organisation (WHO) Patient Safety, WHO, Geneva, Switzerland; 2 Infection Control & WHO Collaborating Centre on Patient Safety, University of Geneva Hospitals, Geneva, Switzerland

## Background

Public health campaigning has been used for a number of decades to promote awareness and understanding about health issues and to mobilize action. In 2015, billions of people across the world have the opportunity to access and support campaigns due to the ownership of smart, mobile technologies. The WHO SAVE LIVES: Clean Your Hands campaign is in its 7th year. In order to maximize the impact of this global annual campaign, each year a new creative focus is given to the topic of hand hygiene in health care.

## Objectives

a) To visually represent real life, using a three-pronged advocacy approach for hand hygiene improvement, with a focus on health-care managers, health workers and patients and using a low budget, engaging and easily replicable idea; b) To stimulate activism across the globe; c) To progress the campaign’s legacy and 2015 theme of strong health systems using hand hygiene as the ‘entrance door’ to infection control.

## Methods

Current global health campaigns featured on social media were reviewed to determine the state of the art and what might best capture people’s attention, aiming to keep the campaign fresh, relevant and connected to the audience’s frame of reference. Hashtags appropriate for use in promoting hand hygiene on a global scale were explored. Communications expertise and a creative agency was secured to further explore and assess the impact of a new medium for promoting the campaign and hand hygiene in health care.

## Results

A one-minute video was created using hospital management staff, health workers and patients, filmed in a real life setting. The focus was on promoting ‘safe hands’ in health care using the phrases ‘I promote’, ‘I provide’ and ‘I deserve’. The video built upon WHO recommendations and previous messages to ensure greatest impact, consistency and credibility.

## Conclusion

Short advocacy videos featuring a promotional concept that people all over the globe can be part of are currently one effective way to promote awareness and understanding of a key public health issue and overcome campaign fatigue.

## Disclosure of interest

None declared.

